# To do or not to do: Large-dose steroid treatment for severe vision loss secondary to compressive inflammatory optic neuropathy in the setting of invasive fungal sinusitis

**DOI:** 10.1016/j.ajoc.2024.102183

**Published:** 2024-10-05

**Authors:** Charissa H. Tan, Benjamin I. Meyer, Colin Kim, Mohammed Raja, Jaylou M. Velez Torres, Jordan Colson, Sander R. Dubovy, Hong Jiang, Byron L. Lam

**Affiliations:** a Bascom Palmer Eye Institute, University of Miami Miller School of Medicine, Miami, FL, 33136, USA; b The Florida Lions Ocular Pathology Laboratory, Bascom Palmer Eye Institute, University of Miami Miller School of Medicine, Miami, FL, 33136, USA; c University of Miami Miller School of Medicine, Miami, FL, 33136, USA; d Transplant Infectious Diseases, Department of Medicine, University of Miami Miller School of Medicine, Miami, FL, 33136, USA; e Department of Pathology and Laboratory Medicine, University of Miami Miller School of Medicine, Miami, FL, 33136, USA; f Department of Neurology, University of Miami Miller School of Medicine, Miami, FL, 33136, USA

**Keywords:** Invasive fungal sinusitis, Compressive optic neuropathy, Inflammatory optic neuropathy, Aspergillus, Mucorales, Large-dose steroid

## Abstract

**Purpose:**

Invasive fungal sinusitis (IFS) is associated with high rates of morbidity and mortality and often presents with orbital apex syndrome. Prompt diagnosis and management are crucial to prevent irreversible visual loss. We report a case of an immunosuppressed patient with rapidly progressive severe visual loss associated with frontal lobe cerebritis and leptomeningitis related to IFS, causing an adjacent compressive inflammatory optic neuropathy, which was treated successfully by large-dose corticosteroids.

**Observations:**

A 29-year-old woman with acute myeloid leukemia status post chemotherapy presented with right-sided headaches and periorbital swelling. Her examination was significant for subjective red desaturation and trace right eyelid edema and ptosis. The remainder of her initial ocular examination was normal. Her labs demonstrated neutropenia and thrombocytopenia. Imaging of the brain and orbits was concerning for extensive sinus disease with intracranial extension. An urgent multi-sinus and optic nerve decompression was performed given concern for compressive optic neuropathy, and the biopsy was consistent with invasive fungal infection. Despite aggressive antifungal treatment, vision in her right eye decreased rapidly to counting fingers. No optic nerve abnormalities were observed on serial MRIs, but adjacent inferior frontal lobe enhancement was present. After a vigorous debate in a multidisciplinary meeting, her severe vision loss was attributed to cerebritis causing an adjacent compressive inflammatory optic neuropathy, and large-dose intravenous (IV) steroid treatment was initiated while maintaining systemic antifungal therapy. Remarkably, she had a full recovery of her vision.

**Conclusions and importance:**

Severe vision loss in IFS can occur due to compressive inflammatory optic neuropathy without direct fungal invasion as a contributing factor. Timely and effective intervention is crucial in preventing vision loss. Large-dose steroid therapy may be a potential treatment option for immunocompromised patients with invasive fungal sinusitis and intracranial invasion, provided strict fungal infection control measures are in place.

## Claim of priority

After conducting a literature review on June 3, 2024 utilizing PubMed using the key words invasive fungal sinusitis and optic neuropathy, we did not find any prior reports of cerebritis from IFS in an immunocompromised patient on chemotherapy causing an adjacent inflammatory optic neuropathy, without direct intraorbital or apical disease.

## 1 Introduction

Invasive fungal sinusitis (IFS) results from colonization of the nasal mucosa by fungal organisms with subsequent spread into adjacent structures, such as the sinuses.^1^^,^^2^ Patients with IFS are typically immunocompromised and classically present with rapidly progressive, painful orbital apex syndrome due to its proximity to the paranasal sinuses. Patients often experience subsequent severe visual impairment due to optic nerve or pathway infarction, ischemia, inflammation, necrosis, and direct invasion.^3^ Prompt diagnosis and appropriate management are crucial to prevent irreversible visual damage and mortality, which is reported between 18 and 88.5 %, with an average overall mortality rate of 40–50 %.^4^, ^5^, ^6^, ^7^, ^8^, ^9^ We present, to our knowledge, the first case report of cerebritis from IFS in an immunocompromised patient on chemotherapy causing an adjacent inflammatory optic neuropathy, without direct intraorbital or apical disease. Remarkably, our patient had a full recovery of her vision and, in fact, resumed chemotherapy and remains in remission at 18 months following steroid treatment.

## 2 Case report

A 29-year-old woman with a recent diagnosis of acute myeloid leukemia (AML), status post induction cytarabine, daunorubicin, and midostaurin and three cycles of consolidation high-dose cytarabine chemotherapy, presented to the emergency department with four days of right-sided headaches, right periorbital swelling, and blurry vision. She had no prior ocular history, surgeries, or relevant family history and had no new environmental exposures. Her initial ocular exam showed trace upper eyelid edema and ptosis. The patient subjectively noted a 10 % red desaturation OD. The remainder of the examination was unremarkable with best corrected visual acuity (BCVA) of 20/20 OU, 11/11 color plates, equal and reactive pupils, no relative afferent pupillary defect (RAPD), intact extraocular movements, and normal fundus. Her neurologic exam was non-focal.

**Fig. 1:**
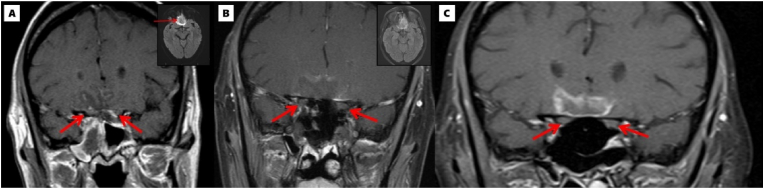
A) Brain MRI with GAD on presentation demonstrates corresponding restricted diffusion (inset) and leptomeningeal enhancement and regional sulcal effacement of the bilateral medial inferior frontal lobes including the gyrus recti. Mild dural thickening and enhancement in bilateral anterior cranial fossa were present. Red arrows showing optic nerves. B) Orbit MRI with GAD one day after right medial orbital, right optic nerve, and multi-sinus decompression demonstrates a similar appearing high FLAIR signal (inset) within the bilateral inferior frontal lobes including the gyrus recti with persistent restricted diffusion and leptomeningeal enhancement. Red arrows showing optic nerves. C) Orbit MRI with GAD after IV methylprednisolone for 3 days followed by prednisone 60 mg three times a day for 10 days demonstrates a significant decrease in the size of the area of abnormal signal involving bilateral inferior medial frontal lobes. Red arrows showing optic nerves. (For interpretation of the references to color in this figure legend, the reader is referred to the Web version of this article.)

Complete blood count revealed neutropenia (total white blood cells: 1.1 × 10^3^/μL with 0.85 × 10^3^/μL absolute neutrophils) and thrombocytopenia (platelet count: 13 × 10^3^/μL). Her neurologic examination was normal. Brain MRI with contrast demonstrated bilateral inferior frontal lobe cerebritis, leptomeningitis, and extensive paranasal sinus disease (Fig. 1A). A subsequent dedicated orbital MRI with and without contrast did not demonstrate optic nerve enhancement (Fig. 1B). Given her immunocompromised status and concern for IFS, she was started on broad-spectrum antifungal treatment consisting of liposomal amphotericin B 5 mg/kg daily and voriconazole (loading dose of 6 mg/kg twice daily followed by 4mg/kg twice daily as maintenance), and the otolaryngology service performed right medial orbital and optic nerve decompression, multi-sinus decompression, and biopsy. Intraoperatively, the bilateral posterior ethmoid and sphenoid sinuses were noted to have dusky mucosa, and detached fungal hyphae were present on frozen section, supporting IFS. A lumbar puncture was deferred due to her thrombocytopenia.

**Fig. 2:**
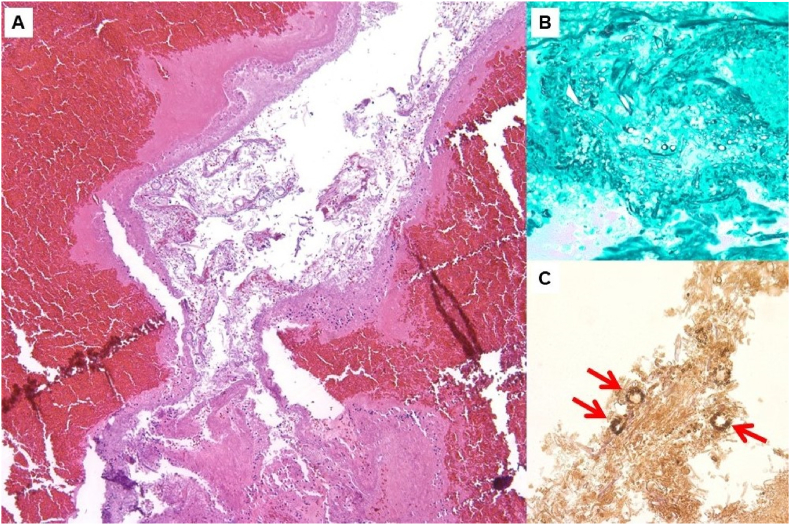
Biopsy obtained during right medial orbital, right optic nerve decompression, and multi-sinus decompression: A) Examination discloses right posterior ethmoid with tissue necrosis (H&E, original magnification ×100). B) Grocott's methenamine silver stain highlights invasive fungal elements (original magnification ×400). C) Immunohistochemistry for Aspergillus highlights a subset of fungal organisms (arrows). The narrow septate hyphae with occasional fruiting bodies are morphologically consistent with Aspergillus species (original magnification ×200).

The patient was switched from voriconazole to posaconazole for broader anti-fungal coverage, and amphotericin B was increased to 7.5 mg/kg daily. However, despite aggressive treatment, she had rapidly progressive vision decline. One week after starting broad-spectrum antifungal medication, the patient's vision declined to 20/20–3 with 10.5/11 color plates OD. The patient was closely followed, with serial complete ophthalmologic examinations daily over the next four days disclosing visual acuities OD of 20/40, then 20/200, then counting fingers only with loss of color vision and interval development of a right RAPD. On each exam, the optic nerve appeared normal without edema, and extraocular motility remained full and painless. Multiple interval MRIs demonstrated stable intracranial findings, without optic nerve enhancement. The biopsy confirmed fungal elements morphologically and immunohistochemically consistent with invasive Aspergillus species (Fig. 2). DNA sequencing later revealed there to be *Rhizomucor miehei* as well. Given the patient's worsening visual acuity, her antifungal regimen was escalated with the addition of IV micafungin 150 mg daily and oral terbinafine 500 mg every 8 h.

After consultation and evaluation with neuro-ophthalmology and neurology, the patient was diagnosed with inflammatory right prechiasmatic optic neuropathy from abutting inferior frontal lobe cerebritis due to the IFS. A multidisciplinary case discussion was held given various complex factors at play. In the context of immunocompromise from chemotherapy, invasive fungal sinus disease with intracranial extension, reactive intracranial inflammation, and inflammatory and compressive optic neuropathy, the decision was made to cautiously start the patient on large-dose steroids, concurrent with her intravenous antifungal therapy and intranasal amphotericin rinses. She received IV 500 mg methylprednisolone with rapid restoration of her vision to 20/25 overnight after one dose. She continued IV methylprednisolone for a total of three days and transitioned to oral prednisone 60 mg daily for 10 days with improvement of her vision back to 20/20, corroborated by improved MRI findings (Fig. 1C). While on steroid treatment, she continued her systemic antifungal regimen. She underwent a total of five sinus washouts, with the ultimate biopsy demonstrating no evidence of fungal elements. Her anti-fungal regimen was de-escalated to posaconazole monotherapy.

**Fig. 3:**
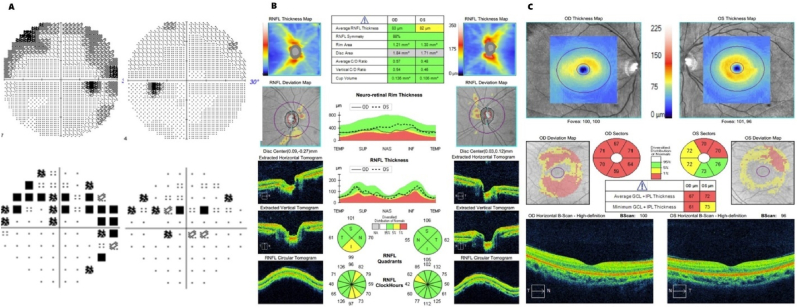
A) 30-2 Humphrey visual field, B) RNFL, and C) GCC at five days after hospital discharge.

**Fig. 4:**
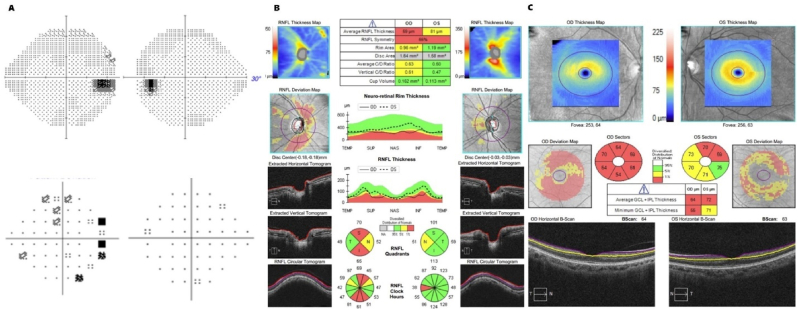
24-2 A) Humphrey visual field, B) RNFL, and C) GCC at 18 months after hospital discharge.

Upon discharge, she was seen in the neuro-ophthalmology clinic, where testing demonstrated nonspecific losses OD > OS on a central 30-2 Humphrey visual field test, retinal nerve fiber layer (RNFL) thickness of 83 μm OD and 82 μm OS, and ganglion cell complex (GCC) thickness of 67 μm OD and 72 μm OS (Fig. 3). Prednisone was tapered over the subsequent four weeks. After three months of stability without decompensation or progressive sinus changes, a repeat bone marrow biopsy demonstrated complete remission of AML with negative minimal residual disease by flow cytometry and next-generation sequencing. She restarted midostaurin maintenance chemotherapy for one year and continued planned concurrent systemic antifungal coverage with posaconazole during the entire length of midostaurin maintenance. On her last follow-up visit 18 months after diagnosis, her condition was stable, with visual acuity 20/20 OU, no RAPD, improved visual field testing, and stable RNFL/GCC loss OD > OS with an RNFL thickness of 59 μm OD and 81 μm OS and GCC thickness of 64 μm OD and 72 μm OS (Fig. 4).

## 3 Discussion

IFS, such as from *Aspergillus* or *Mucorales* species, as observed in our patient, can cause compressive and inflammatory optic neuropathy. Since the fungal infection can spread via erosion of bone and/or through the vasculature, both orbital and intracranial extension are not uncommon, as observed in our patient.^10^ Invasion of the orbit by the offending pathogen can cause swelling of the face, ophthalmoplegia, proptosis, pain, and vision loss, while extension into the intracranial space can lead to cerebritis or meningitis.^1^^,^^11^ A previous case by Hasan et al. described an episode of optic neuritis following encephalitis, suggesting that inflammation of the optic nerve may be a rare sequelae of inflammation of the brain.^12^ Similarly, the repeated MRIs of our patient showed inferior frontal lobe abnormal enhancement with no abnormal signals of optic nerves, suggesting that the patient's visual loss might be due to compressive inflammatory optic neuropathy secondary to adjacent cerebritis affecting the contiguous optic nerve. However, possible optic neuritis due to fungal invasion and related inflammation could not be completely excluded because, although enhancement on MRI is sensitive for detecting optic nerve involvement secondary to acute fungal invasion, up to 6 % of cases may not have enhancement of the optic nerves on MRI.^3^^,^^13^

Once the fungal infection involves the central nervous system (CNS), it has a mortality rate of up to 100 %.^14^^,^^15^ The fatality rate depends on patient demographics, their underlying conditions, the site of infection, the treatment, and the degree of neutropenia.^14^ Our patient had unfavorable prognostic factors, including hematologic malignancy, CNS involvement, and neutropenia.^16^^,^^17^ Patients with an absolute neutrophil count of less than 500 were more likely to have a fatal outcome in several studies, which is thought to result from the decrease of reactive oxygen intermediates and antimicrobial peptides generated by immune cells.^4^^,^^7^^,^^8^ As a result, reversing the underlying immunosuppression present in most IFS cases with leukocyte infusions, granulocyte-macrophage colony-stimulating factor, and other immunomodulating agents have been explored with mixed results.^16^^,^^18^

Conversely, causing immunosuppression with corticosteroids in conjunction with antifungal therapy in the setting of IFS is controversial. Corticosteroid therapy predisposes individuals to invasive fungal infections of the sinus, particularly in those who are immunocompromised, similar to our patient.^19^^,^^20^ Treatment of fungal sinusitis, therefore, typically includes surgical debridement and antifungal therapy with avoidance of further immunosuppression.^2^^,^^21^ Use of corticosteroids is also reported to be associated with higher mortality rates in fungal infection.^19^ However, large-dose corticosteroids are the current mainstay treatment for acute inflammatory optic neuropathy.^22^, ^23^, ^24^ A delicate balance between attenuating the host immune system and treatment of the optic nerve inflammation is required. While there are limited reports of using corticosteroids to manage the inflammatory component of IFS, Yamanoi et al. presented a case in which methylprednisolone sodium succinate pulse therapy was used with itraconazole and amphotericin B with rapid improvement of symptoms.^25^ In both their case and ours, there was successful restoration of neurologic function while maintaining infection control, though optic nerve function was still adversely affected in the patients described by Yamanoi et al.

The decision to initiate corticosteroid therapy was made after the patient experienced vision loss from 20/20 to counting fingers over the course of several days. Though it could have been possible that the antifungal treatment had reached a level of efficacy, the patient was deteriorating even while on the treatment regimen for seven days, and her serum voriconazole level was in the target therapeutic range at 2.5 mcg/mL. There is no consensus on the length of IV antifungal treatment required for control of IFS nor on the dosage or duration of steroid therapy used in cases of fungal infections, but given the dire circumstance, large-dose IV corticosteroids were initiated.^16^^,^^21^ It is uncertain why corticosteroid therapy was effective in this case. A possible explanation is that the patient's immune system was already compromised and not effectively combating the infection, so the addition of corticosteroid therapy to reduce inflammation did not substantially affect the patient's immunocompromised status. The patient's absolute neutrophil count, which is a factor that has been demonstrated to improve outcomes, also increased either due to count recovery or from the corticosteroids. Another consideration is leukemic optic neuropathy from AML, which could present similarly and respond well to corticosteroid therapy. However, multiple MRIs showed no abnormal enhancement of the optic nerve. Additionally, her oncologist monitored her closely throughout the course, with no evidence of AML recurrence. Regardless, the patient's vision dramatically improved after IV methylprednisolone, and she achieved complete remission of AML without requiring further treatment. Therefore, initiation of large-dose steroid therapy for inflammatory optic neuropathy in immunocompromised patients with IFS may be a potential treatment option after consideration of risks and benefits.

## 4 Conclusions

The presentation of severe vision loss in the setting of IFS without direct fungal invasion in this case was a rare diagnostic challenge and thought to occur due to compressive inflammatory optic neuropathy. There were no clear treatment options due to the complexity of factors, including the patient's history of AML, invasive *Aspergillus* and *Mucorales*, and rapidly progressive vision loss. Timely and effective intervention is crucial in preventing permanent vision loss and mortality, and our patient not only survived but also recovered to a visual acuity of 20/20. This case demonstrates that large-dose steroid therapy may be a potential treatment option for immunocompromised patients with IFS and intracranial involvement, provided that strict fungal infection control measures are in place.

## Patient consent

This report does not contain any personal information that could lead to the identification of the patient.

## Funding

The Bascom Palmer Eye Institute is supported by NIH Center Core Grant P30EY014801 and a Research to Prevent Blindness Unrestricted Grant (GR004596-1). The Florida Lions Ocular Pathology Laboratory is supported by the 10.13039/100016850Beauty of Sight Foundation.

## Authorship

All authors attest that they meet the current ICMJE criteria for authorship.

## CRediT authorship contribution statement

**Charissa H. Tan:** Writing – review & editing, Writing – original draft, Visualization, Methodology, Investigation, Formal analysis, Data curation, Conceptualization. **Benjamin I. Meyer:** Writing – review & editing, Investigation, Data curation, Conceptualization. **Colin Kim:** Writing – original draft, Investigation. **Mohammed Raja:** Writing – review & editing, Validation, Data curation. **Jaylou M. Velez Torres:** Writing – review & editing, Validation, Data curation. **Jordan Colson:** Writing – review & editing, Validation, Data curation. **Sander R. Dubovy:** Visualization, Resources, Investigation. **Hong Jiang:** Writing – review & editing, Supervision, Project administration, Methodology, Investigation, Conceptualization. **Byron L. Lam:** Writing – review & editing, Supervision, Project administration, Methodology, Investigation, Conceptualization.

## Declaration of competing interest

The authors declare that they have no known competing financial interests or personal relationships that could have appeared to influence the work reported in this paper.

The authors have no conflict of interest.
